# Telerehabilitation of acute musculoskeletal multi-disorders: prospective, single-arm, interventional study

**DOI:** 10.1186/s12891-021-04891-5

**Published:** 2022-01-04

**Authors:** Fabíola Costa, Dora Janela, Maria Molinos, Jorge Lains, Gerard E. Francisco, Virgílio Bento, Fernando Dias Correia

**Affiliations:** 1 SWORD Health Technologies, Inc, Draper, UT USA; 2 Rovisco Pais Medical and Rehabilitation Centre, Tocha, Portugal; 3 Faculty of Medicine, Coimbra University, Coimbra, Portugal; 4 Department of Physical Medicine and Rehabilitation, The University of Texas Health Science Center McGovern Medical School, and TIRR Memorial Hermann, Houston, TX USA; 5 Neurology Department, Centro Hospitalar e Universitário do Porto, Porto, Portugal

**Keywords:** Musculoskeletal pain, Home-based digital rehabilitation, Digital therapy, Physical therapy, eHealth, Rehabilitation

## Abstract

**Background:**

Acute musculoskeletal (MSK) pain is very common and associated with impaired productivity and high economic burden. Access to timely and personalized, evidence-based care is key to improve outcomes while reducing healthcare expenditure. Digital interventions can facilitate access and ensure care scalability.

**Objective:**

Present the feasibility and results of a fully remote digital care program (DCP) for acute MSK conditions affecting several body areas.

**Methods:**

Interventional single-arm study of individuals applying for digital care programs for acute MSK pain. Primary outcome was the mean change between baseline and end-of-program in self-reported Numerical Pain Rating Scale (NPRS) score and secondary outcomes were change in analgesic consumption, intention to undergo surgery, anxiety (GAD-7), depression (PHQ-9), fear-avoidance beliefs (FABQ-PA), work productivity (WPAI-GH) and engagement.

**Results:**

Three hundred forty-three patients started the program, of which 300 (87.5%) completed the program. Latent growth curve analysis (LGCA) revealed that changes in NPRS between baseline and end-of-program were both statistically (*p* < 0.001) and clinically significant: 64.3% reduction (mean − 2.9 points). Marked improvements were also noted in all secondary outcomes: 82% reduction in medication intake, 63% reduction in surgery intent, 40% in fear-avoidance beliefs, 54% in anxiety, 58% in depression and 79% recovery in overall productivity. All outcomes had steeper improvements in the first 4 weeks, which paralleled higher engagement in this period (3.6 vs 3.2 overall weekly sessions, *p* < 0.001). Mean patient satisfaction score was 8.7/10 (SD 1.26).

**Strengths and limitations:**

This is the first longitudinal study demonstrating the feasibility of a DCP for patients with acute MSK conditions involving several body areas. Major strengths of this study are the large sample size, the wide range of MSK conditions studied, the breadth of outcomes measured, and the very high retention rate and adherence level. The major limitation regards to the absence of a control group.

**Conclusions:**

We observed very high completion and engagement rates, as well as clinically relevant changes in all health-related outcomes and productivity recovery. We believe this DCP holds great potential in the delivery of effective and scalable MSK care.

**Trial registration:**

NCT, NCT04092946. Registered 17/09/2019;

**Supplementary Information:**

The online version contains supplementary material available at 10.1186/s12891-021-04891-5.

## Introduction

Acute MSK injuries are very common, particularly among working-age adults, with 2.9 million injuries in the workplace reported in 2016 [1, 2], as well as in sports, with ~ 4.2 million emergency room visits between 2014 and 2016 in the United States (US) [2]. The combination of high incidence and both high direct (diagnosis, initial management, rehabilitation) and indirect costs (lost work productivity, lost wages for workers) translates into a very high economic burden [3].

Importantly, the onset of MSK pain can result in a downward spiral of negative physical, social, and psychological consequences, which can lead to chronic MSK pain [4–9]. It has been reported, for example, that about 31% of patients do not fully recover from low back pain within 6 months [10] and that 45 to 55% of acute knee injuries develop into a long-term medical condition [11].

Persistent painful stimuli are believed to provide a background for the development of chronic pain [12], and the transition of acute to chronic pain appears to involve peripheral and central sensitization [8, 13, 14], deconditioning from fear-avoidance and other maladaptive behaviors, and a host of other psychosocial factors [15]. Providing effective interventions on the acute and subacute stages to prevent chronification is therefore a priority [16, 17]. Since numerous studies have demonstrated that depression, anxiety and other psychological co-morbidities (e.g. catastrophization, poor coping skills) are risk factors for pain chronification [7, 12, 18], there is also a need to address these aspects.

Balancing comfort and safety following acute MSK injury is possible when using a multimodal approach combining pharmaceutical, cognitive, and physical strategies [19]. Clinical data has demonstrated the benefit of exercise on functional outcomes and long-term re-injury prevention following acute MSK conditions [20–22]. Early intervention has been associated with significant improvements at lower cost, while preventing long-term disability [6, 23].

However, access to timely interventions remains a challenge, due to limitations in the availability of physical therapists, physical mobility, transportation and/or costs [24–27]. Also, compliance with physical therapy, a key determinant to therapy success [28, 29] is poor, with studies reporting non-compliance or dropout rates greater than  50% [30–34]. Telerehabilitation has shown to provide comparable results to outpatient physical therapy [35–39] and to face-to-face home rehabilitation [40, 41], while reducing costs [42–44]. There is, however, a lack of evidence on its applicability in acute MSK conditions other than after surgery. Furthermore, this approach requires real-time availability of physical therapists (PT), hence the need for scalable solutions that enable asynchronous care delivery.

We have developed a digital care program (DCP) delivered through a tablet, motion trackers and a cloud-platform. The DCP enables patients to receive a program tailored by an assigned PT, having real-time biofeedback during exercises (which are then asynchronously monitored) and a bi-directional communication channel. Moreover, this DCP combines a multimodal approach, integrating exercise, education and cognitive behavioral therapy (CBT). This DCP was previously validated (feasibility and effectiveness) in post-surgery MSK rehabilitation [38, 41, 45, 46].

The aim of this study is to describe the feasibility of this DCP on a real-world cohort of patients with acute MSK conditions and the clinical outcomes change. Our hypothesis was that the observed outcomes would be at least similar to those reported in the literature for other digital or conventional therapies.

## Methods

### Study design

This decentralized, interventional, single-arm study evaluated patients at multiple locations in the United States, who were treated with a home-based DCP between September 18th 2020 and March 8th 2021. The study was approved by New England IRB (protocol number 120190313) and prospectively registered in ClinicalTrials.gov (17/09/2019) (NCT04092946), with recruitment starting on the same day. All patients were informed about the purpose and procedures of the study and provided informed consent.

### Participants

Adults (> 18 years old) covered by the health plans of 9 participating employers were invited to apply to SWORD Health’s digital MSK care program through a dedicated website. Participants were included in the study if they reported acute (< 12 weeks) MSK pain in: i) neck; ii) low back; iii) shoulder; iv) elbow; v) hip; vi) knee; or vii) ankle. Exclusion criteria were: a) fracture or significant trauma in the area of pain, including surgery; b) unexpected and rapidly progressive loss of strength or numbness in the limbs in the last 2 weeks; c) unexplained and change of bowel or urinary function in the last 2 weeks; d) active cancer or undergoing treatment for cancer; e) other health condition (e.g. cardiac, respiratory) incompatible with at least 20 min of light to moderate exercise.

To mitigate the risk of selection bias, we included all consecutively qualified participants who enrolled in the DCP until January 8th, 2021.

### Intervention

The DCP consisted of exercise, and education including cognitive behavioral therapy (CBT)-related topics. Upon enrollment, all participants were assigned a physical therapist (PT). Each participant was asked to complete at least 3 exercise sessions a week. These sessions were performed using a class II medical device that uses motion tracking technology to digitize motion and provide real-time biofeedback during exercise. Intervention length varied between 8 and 12 weeks, according to the specific needs of each patient, as assessed by the assigned PT. Participant performance and progress were remotely monitored by the PT through a cloud-based platform. Secure bi-directional communication was ensured through a dedicated smartphone app.

The educational component consisted of educational articles and interactive modules based on CBT topics, including catastrophizing, active coping methods, and fear avoidance, tailored to the participant’s condition, and made available twice per week through the app.

### Outcomes

Primary outcome was the mean change in Numerical Pain Rating Scale (NPRS) score between baseline and end of program, assessed through the question “Please rate your pain over the last 7 days: 0 (no pain at all) to 10 (worst pain imaginable)”. Participants were classified as responders and non-responders according to the minimal clinically important difference (MCID) of 2.0 reported by Childs et al. [47] for the NPRS.

Secondary outcomes were the mean changes between baseline and end of program for:

- Analgesic usage, assessed through the questions: “Are you currently taking any pain medication?” and “If you answered yes, how many times per week are you taking pain medication?”;
- Surgery intent, addressed through the question “How likely are you to have surgery to address your condition in the next 12 months: 0 (not at all likely) to 100 (extremely likely)?”;
- Anxiety, measured by the Generalized Anxiety Disorder (GAD-7) 7-item questionnaire (range 0–21) [48], and depression by Patient Health (PHQ-9) 9-item questionnaire (range 0–27) [49]. A threshold of 5 was used to identify at least mild anxiety or depression;
- Fear-avoidance, assessed through the Fear-Avoidance Beliefs Questionnaire for physical activity (FABQ-PA), composed by a total of 4 items, each with a 7 option Likert scale (range 0–24) [50];
- Impact on productivity, evaluated in working participants by Work Productivity and Activity Impairment (WPAI) questionnaire, including overall work impairment, presenteeism, absenteeism and activities impairment [51];
- Engagement, assessed through the program duration (weeks); number of completed sessions per week; total exercise time (minutes); and overall satisfaction (points), through the question: “On a scale from 0 to 10, how likely is it that you would recommend this intervention to a friend or neighbor?”.

Participants that, at any point, did not engage in any exercise session for 28 consecutive days were considered dropouts.

### Safety and adverse events

Patients were instructed to report any adverse events to their PT through the built-in secure chat feature or telephone call. Additionally, pain and fatigue scores (graduated from 0 to 10) were collected at the end of each session and monitored remotely by the PT.

### Data availability

The data supporting the conclusions of this article is included within the article and its additional files. The rehabilitation protocols, de-identified data and analysis codes may be provided on request to the corresponding author.

### Statistical analysis

To assess differences in clinical and demographic variables between completers and non-completers, responders and non-responders and different body areas, an independent samples t test or a one-way ANOVA with Bonferroni post-hoc was used for quantitative variables and a Chi-squared test for qualitative variables. To assess differences in usability-related outcomes between 4 and 8 weeks a paired-samples t-test was applied. The impact of mental health symptomatology on engagement was also assessed through independent samples t test. Bivariate correlations (Pearson r) were used to investigate associations between outcomes. These analyses were performed using SPSS V.17.0 (SPSS Inc., Chicago, Illinois, USA).

To model the trajectory of outcome variables over time a latent growth curve analysis (LGCA) approach was applied [52, 53]. LGCA is robust to bias due to missing data, through the use of full information maximum likelihood (FIML) estimation [54], which acknowledges that repeated measures on the same individual are correlated [55]. The intercepts represent initial status at baseline for each variable. The slopes represent the expected linear change in the outcome per week as the time score changes [52]. Both are adjusted for covariates and fitted as random effects allowing each to vary between individuals (see structural equation and path diagram for the LGCAs used in Supplementary Fig. 1). All models were estimated with a robust sandwich estimator for standard errors. This analysis was performed both with unfiltered and filtered cases at baseline according to the following thresholds: medication intake > 0, surgery intent > 0 points, GAD-7 ≥ 5 points; PHQ-9 ≥ 5 points; WPAIs scores > 0 points. Dropouts and clinical exclusions were also filtered for the main analysis. Finally, a conditional model was used to assess the influence of age, sex and body mass index (BMI) on recovery trajectories. Estimation of model fit was assessed through chi-squared test, root mean square error of approximation (RMSEA), confirmatory fit index (CFI), and standardized root mean square residual (SRMR) [56, 57]. Significant levels were set at *p* < 0.05 in all analyses. LGCA was coded using R, version 1.4.1717.

## Results

**Fig. 1:**
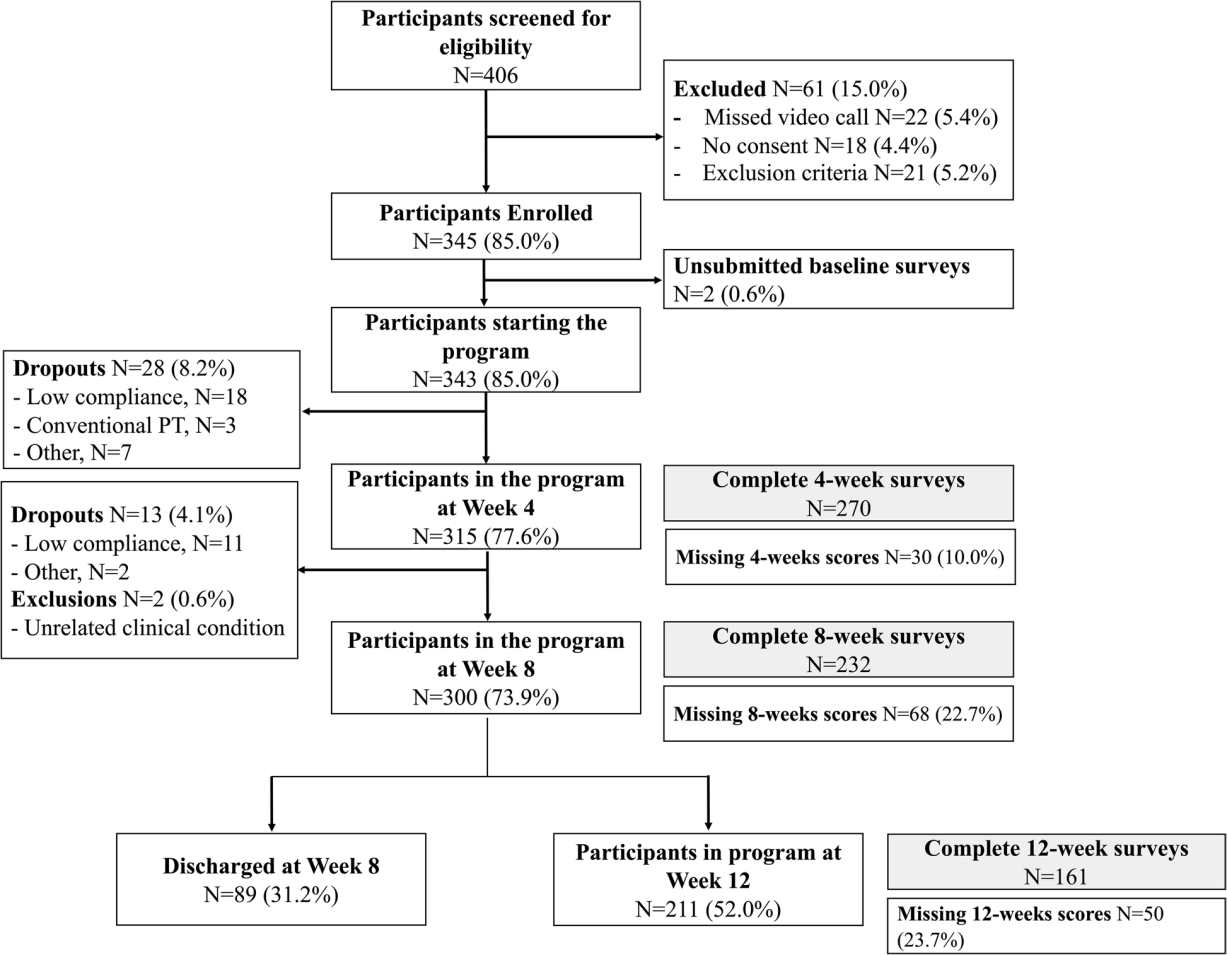
Flow Diagram of the study

A total of 406 participants were screened for eligibility from nine recruitment sites. From these, 5.4% (22/406) applied to the program but missed the enrollment video call, 4.4% (18/406) declined to participate, and 5.2% (21/406) had exclusion criteria, resulting in 345 enrolled participants. The flow diagram for the study is presented in Fig. 1. Program completion rate was 87.5% (300/343).

### Baseline characteristics

Baseline characteristics of participants (*N* = 343) are presented in Table 1. There were no differences in age (*p* = 0.085), sex (*p* = 0.174) or BMI (*p* = 0.295) between different body areas. Also, no significant baseline differences in demographic or clinical characteristics were observed between completers (*N* = 300) and non-completers (*N* = 43), except for baseline exercise levels, which were higher in completers (*p* = 0.017) (Supplementary Table S1). Participants discharged at 8 weeks were slightly younger than those completing the program at 12 weeks (50.1 (SD 11.4) vs 53.0 (SD 10.9) years, *p* = 0.048), with no other significant differences noted (Supplementary Table S2). Clinical outcomes and engagement levels did not differ between these subgroups.

**Table 1:** Baseline characteristics of study participants (*N* = 343)

Characteristic	Estimate
Age (years), mean (SD)	51.1 (11.4)
Age categories, N (%):	
∙ < 25	7 (2.0)
∙ 25–40	65 (19.0)
∙ 40–60	191 (55.7)
∙ > 60	80 (23.3)
Sex, Female, N (%)	205 (59.8)
BMI, mean (SD)	28.8 (6.55)
BMI categories, N (%):	
∙ Underweight (< 18.5)	2 (0.6)
∙ Normal (18.5–25)	113 (32.9)
∙ Overweight (25–30)	114 (33.2)
∙ Obese (30–40)	87 (25.4)
∙ Obese grade III (> 40)	27 (7.9)
Education level^a^, N (%):	
∙ High	286 (83.4)
∙ Low	29 (8.4)
∙ Undisclosed	28 (8.2)
Employment status, N (%):	
∙ Employed (part-time or full-time)	303 (88.3)
∙ Unemployed (not working or seeking for opportunities)	40 (11.7)
Occupation type, N (%):	
∙ White collar	298 (86.9)
∙ Blue collar	29 (8.5)
∙ Other (e.g. retired)	16 (4.7)
Affected body area, N (%):	
∙ Ankle	23 (6.7)
∙ Elbow	27 (7.9)
∙ Hip	35 (10.2)
∙ Knee	61 (17.8)
∙ Low back	94 (27.4)
∙ Neck	30 (8.7)
∙ Shoulder	73 (21.3)
Exercise level, days per week, N (%):	
∙ None	54 (15.7)
∙ 1–2 days	187 (54.5)
∙ 3–4 days	102 (29.7)
Duration of pain, N (%):	
∙ < 4 weeks	108 (31.5)
∙ 4–12 weeks	235 (68.5)

^a^High: college/University; Low: professional school or high school; *BMI* Body mass index

Participant distribution stratified by body area (Supplementary Fig. 2), did not differ between the four time points assessed.

### Clinical outcomes

**Fig. 2:**
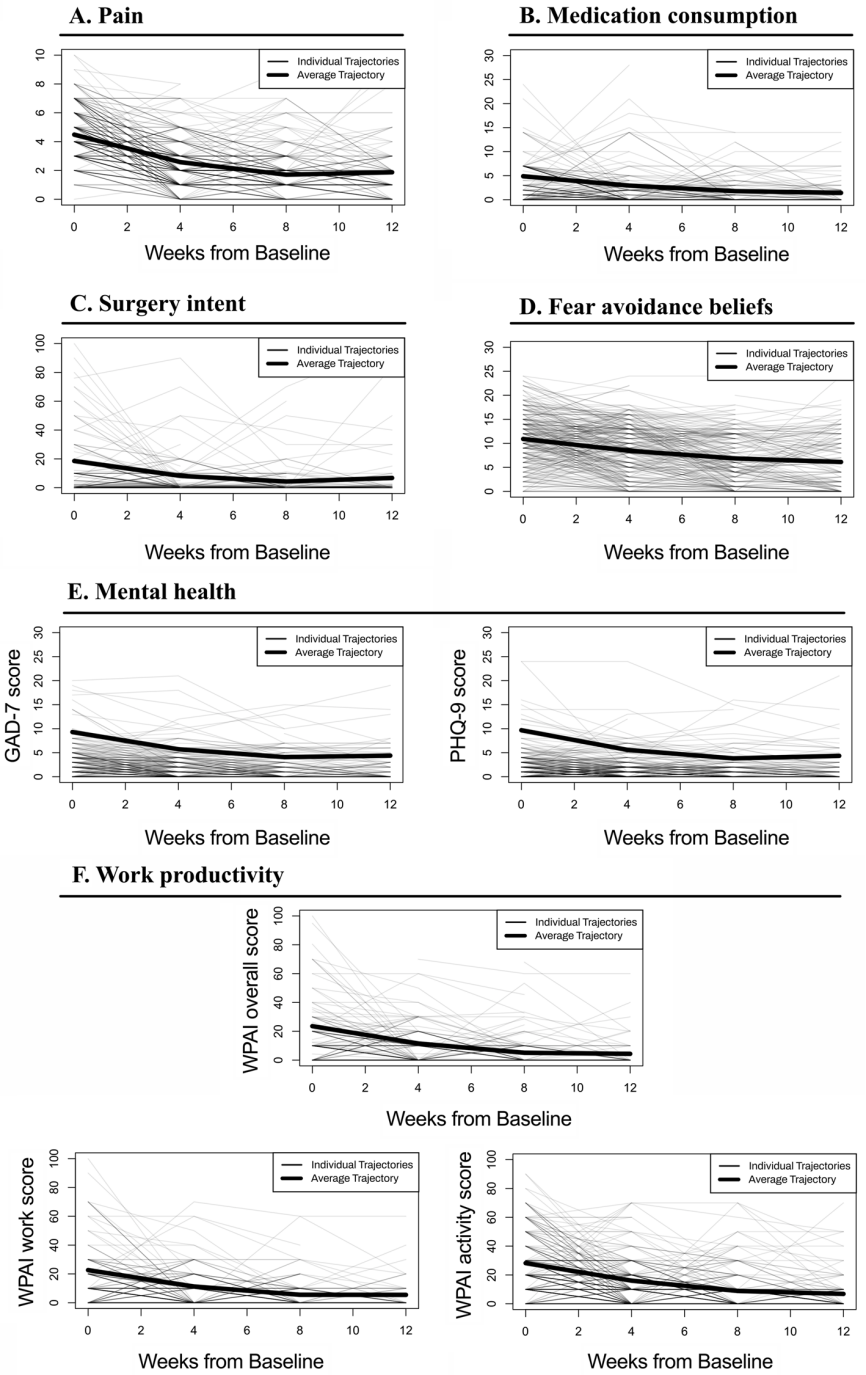
Longitudinal changes across time for all filtered variables: **A**: Pain; **B**: Medication consumption; **C**: Surgery Intent; **D**: Fear-avoidance beliefs; **E**: Mental health (GAD-7 and PHQ-9); **F**: Work productivity (WPAI overall, WPAI work, WPAI activity). Cases filtered according to the following baseline thresholds - medication usage per week > 0 days; surgery intent scores > 0 points; GAD-7 scores ≥ 5 points; PHQ-9 scores ≥ 5 points; all WPAI > 0 points

Longitudinal changes in outcome variables were assessed for program completers (*N* = 300), through LGCA using 2 models: unconditional and conditional, i.e., with age, sex and BMI as control variables, both for filtered and unfiltered data. Results of the unconditional model are represented in Table 2, where intercept represents scores at baseline, slope represents change over time (weeks) and curve estimates possible leveling effect towards the end of the trajectory. Trajectories for each outcome are depicted in Fig. 2, and the main findings of this analysis are summarized throughout the text. Model fit assessments for each variable are presented in Supplementary Table S3. Results of the conditional model are detailed in Supplementary Table S4 and discussed throughout the text as relevant.

**Table 2:** Results from the unconditional Latent Growth Curve analysis

Outcome	Intercept		Slope		Curve	
	Mean (SD)	***p***	Mean (SD)	***p***	Mean (SD)	***p***
Pain	4.48 (1.41)	**<.001**	−0.6 (0.44)	**<.001**	0.03 (0.03)	**<.001**
Medication Usage > 0	5.13 (3.78)	**<.001**	−0.83 (1.02)	**<.001**	0.04 (0.05)	**<.001**
Medication Usage (all)	2.10 (3.65)	**<.001**	−0.14 (0.85)	0.092	0.003 (0.03)	0.599
Surgery Intent > 0	18.52 (15.44)	**<.001**	−3.36 (4.08)	**<.001**	0.20 (0.35)	**0.001**
Surgery Intent (all)	5.82 (10.98)	**<.001**	−0.95 (2.54)	**<.001**	0.06 (0.20)	**0.002**
GAD ≥5	9.30 (3.77)	**<.001**	−1.14 (0.86)	**<.001**	0.06 (0.03)	**<.001**
GAD (all)	2.81 (3.48)	**<.001**	−0.26 (0.62)	**<.001**	0.01 (0.03)	**<.001**
PHQ ≥5	9.69 (4.66)	**<.001**	−1.31 (1.14)	**<.001**	0.07 (0.06)	**<.001**
PHQ (all)	2.40 (3.26)	**<.001**	−0.17 (0.66)	**<.001**	0.007 (0.04)	**0.049**
FABQ	10.91 (4.59)	**<.001**	−0.72 (0.85)	**<.001**	0.03 (0.05)	**<.001**
WPAI Overall > 0	23.55 (11.63)	**<.001**	−3.71 (3.65)	**<.001**	0.18 (0.22)	**<.001**
WPAI Overall (all)	10.89 (13.05)	**<.001**	−1.15 (3.09)	**<.001**	0.04 (0.17)	**0.044**
WPAI Work Impairment > 0	22.41 (10.84)	**<.001**	−3.44 (3.34)	**<.001**	0.16 (0.20)	**<.001**
WPAI Work Impairment (all)	10.23 (11.86)	**<.001**	−1.03 (2.90)	**<.001**	0.04 (0.14)	0.078
WPAI Activity Impairment > 0	28.20 (9.18)	**<.001**	−3.67 (2.99)	**<.001**	0.16 (0.22)	**<.001**
WPAI Activity Impairment (all)	21.56 (13.08)	**<.001**	−2.50 (2.89)	**<.001**	0.10 (0.20)	**<.001**

### Pain

The mean overall change as estimated by the LCGA model was 2.88 points (95% CI − 0.25 to 3.96), corresponding to a 64.3% improvement from baseline (Table 2). Pain levels decreased over time (*p* < 0.001, Fig. 2 and Table 2), at an average of 0.60 points per week (SD 0.44). The decline was steeper from baseline to 4 weeks (Fig. 2). Females reported higher pain at baseline, but recovery trajectories were not influenced by this or other parameters (Supplementary Table S4).

Of note, 74.8% (187/250) of completers with available scores at end of program improved by at least 2 points (responders). Non-responders presented lower pain levels at baseline (3.3, SD 1.6 vs 4.9, SD 1.6, *p* < 0.001), which may explain smaller pain change, and were older (55.5, SD 9.3 vs 50.8, SD 11.3 years, *p* = 0.002) (Supplementary Table S5). Even though pain trajectories declined for all body areas, there was a lower percentage of hip and low back patients and higher percentage of neck patients among non-responders (Supplementary Table S5).

### Medication

Pain killers’ consumption decreased over time (*p* < 0.001, Table 2 and Fig. 2), at an average of 0.83 points per week (SD 1.02), corresponding to an average reduction of 81.9% (mean difference 4.20 points, 95% CI − 5.74 to 8.96). Baseline characteristics had no influence on pain killers’ intake (Supplementary Table S4).

Within completers with available scores at end of program (*N* = 250), 43.6% were taking medication at baseline, in comparison to 37.0% at end of program.

### Surgery intent

Surgery intent decreased over time, (*p* < 0.001, Table 2), with an average reduction of − 0.95 points (SD 4.08) per week, corresponding to a 62.7% reduction (11.6 points, 95% CI − 28.23 to 41.56) by end of program. Participants with higher BMI had both higher surgery intent (intercept 1.03, *p* < 0.001), and steeper decline of surgery intent (− 0.29 intention per week (*p* = 0.030), Supplementary Table S4). No other covariates impacted surgery intent (Supplementary Table S4). The decrease in surgery intent was correlated with pain recovery (r(250) = 0.149, *p* = 0.019).

### Productivity

Absenteeism was low at baseline, with an average of 7.5% (18/239) participants missing work time in the previous week. Presenteeism was more prevalent, with 45.2% (108/239) participants reporting an average of 22.41 (SD 10.34) impairment in productivity (Table 2). Presenteeism decreased during the DCP (*p* < 0.001), with an average reduction of − 3.44 (SD 3.34) per week, corresponding to an overall 81.4% reduction until the end of the program (Table 2 and Fig. 2). Older participants recovered at a faster pace (− 0.17 average reduction per week) (*p* = 0.007) with leveling off effect towards the end of the intervention (*p* = 0.01) (Supplementary Table S4). No other covariates had influence on presenteeism (Supplementary Table S4). A moderate correlation was observed between presenteeism recovery and pain reduction (r(76) = 0.362, *p* = 0.001).

There was a decrease in overall productivity impairment (absenteeism+presenteeism) over time (*p* < 0.001, Table 2) with an average reduction of 79.0%, (mean change 18.60 points, 95% CI − 28.81 to 37.48). Productivity recovery was correlated with pain reduction (r(77) = 0.382, p = 0.001).

Additionally, 46.0% (110/239) of individuals reported some degree of non-work related activity impairment at baseline (intercept 28.20, SD 9.18), with significant improvement over time (*p* < 0.001, Table 2) and a reduction of 74.5% at end of program (mean change 21.00 points, 95% CI − 15.08 to 28.76), *p* < 0.001). Moderate correlation was observed between recovery of activity impairment and pain reduction (r(192) = 0.430, *p* < 0.001).

### Mental health and fear-avoidance

Average FABQ-PA scores at baseline were moderate (10.91, SD 4.59), with 20% (60/300) participants presenting with an FABQ-PA score above 15 points (18.84, SD 2.44), a threshold that has been associated with poorer prognosis [8, 12]. FABQ-PA scores decreased over time (*p* < 0.001, Table 2), with a 39.5% reduction at end of program (mean difference 4.32 points, 95% CI 0.93 to 11.3, *p* < 0.001), and 83.3% (40/48) of the participants who scored above 15 at baseline ending the program below this score. FABQ-PA reduction was correlated with pain reduction (r(240) = 0.238, *p* < 0.001) and with recovery in activities impairment (r(89) = 0.369, *p* < 0.001).

A low number of participants screened positive for anxiety and depression, with 23.3% (70/300) self-reporting a GAD-7 score ≥ 5 and 15.0% (45/300) a PHQ-9 score ≥ 5, which was not surprising considering the acute nature of the MSK condition. Participants screening positive for anxiety at baseline improved over time (*p* < 0.001, Table 2), with an average reduction of 54.2% at end of program (mean difference 5.04 points, 95% CI − 4.34 to 13.14, *p* < 0.001). Similarly, participants screening positive for depression at baseline also improved over time (*p* < 0.001, Table 2), with a 58.2% reduction at end of program (mean difference 5.64 points, 95% CI − 8.67 to 17.38, *p* < 0.001). These recovery paths were not influenced by covariates (Supplementary Table S4). A strong correlation was observed between anxiety reduction and change in presenteeism (r(20) = 0.570, *p* = 0.009), as well as between pain reduction and depression reduction (r(33) = 0.360, *p* = 0.040).

### Usability and engagement

Usability and engagement metrics are summarized in Table 3. Participants performed on average 3.2 sessions per week (SD 1.29), with 53.7% (161/300) performing more than the recommended 3 sessions/week. Weekly engagement did not differ between body area (*p* = 0.102) and no associations were found with demographic characteristics. Mean total exercise dosage was 422.9 min (SD 198.4) and 555.9 min (SD 284.1) for 8 and 12-week completers, respectively. Engagement was higher in the first 4 weeks as compared to 8 weeks (mean 3.6 weekly sessions (SD 1.4) versus 3.4 sessions, (SD 1.3), *p* < 0.001). Average NRS fatigue and pain levels during sessions were low (< 2 points) and decreased slightly over time (*p* = 0.003 and *p* < 0.001, respectively). Regarding the educational component, participants read on average 1.5 (SD 0.9) articles per week. Overall satisfaction level was high (8.7/10) and increased over time (*p* < 0.001).

**Table 3:** Usability and engagement in all participants

Usability outcomes Mean (SD)	At study end (***N*** = 300)	4 weeks (***N*** = 300)	8 weeks (***N*** = 300)	12 weeks (***N*** = 211)
Total number of sessions	–	13.9 (5.92)	25.6 (11.27)^#^	34.7 (17.29)
Total exercising time, minutes	–	231.9 (105.37)	422.9 (198.41)^#^	555.9 (284.10)
Number of sessions per week	3.2 (1.29)	3.6 (1.42)	3.4 (1.33)^#^	3.2 (1.32)
Average pain during sessions (NRS, 0–10)	1.4 (0.92)	1.7 (1.06)	1.5 (0.97)^#^	1.4 (0.94)
Average fatigue during sessions (NRS, 0–10)	1.2 (1.09)	1.3 (1.24)	1.3 (1.15)^#^	1.2 (1.05)
Average satisfaction (NRS, 0–10)	8.7 (1.26)	8.7 (1.26)	8.8 (1.18)^#^	8.8 (1.21)
Educational articles per week	1.5 (0.90)	–	**–**	–

^#^Estimates at 8 weeks were significantly different from those at 4 weeks as per the paired samples t-test, *P* < 0.001 for average pain and *P* = 0.003 for average fatigue

Interestingly, patients with depressive symptoms (PHQ-9 ≥ 5, *N* = 45/300) performed less sessions overall (*p* = 0.004), which translated into less total exercising time (*p* < 0.001), while also reporting higher pain and fatigue levels during sessions (*p* < 0.001 and *p* = 0.015, respectively) (Supplementary Table S6). Patients with anxiety at start (GAD-7 ≥ 5, *N* = 70/300) also spent less time exercising (*p* = 0.008) and reported higher pain levels at session end (*p* < 0.001) as compared to non-anxious patients.

## Discussion

### Main findings

The DCP presented herein was able to promote very high patient engagement and compliance rates, thus demonstrating its feasibility in a large real-world cohort of patients with acute MSK conditions. We observed great reduction in pain levels (mean change 64.3%), paralleled with a high decrease in pain killer intake (81.9%). Also, surgery intent, which is one of the strongest predictors of future surgery [58, 59], was markedly reduced (62.7%), in line with literature favoring non-surgical management of MSK conditions [60]. Marked reductions in anxiety, depression (54.2 and 58.2%, respectively) and fear-avoidance behaviors (39.5% reduction) were also observed. Both the improvement in pain and in mental health correlated with productivity improvement, opening new avenues of research about the pertinence of multimodal DCP in the reduction of the economic burden associated with these conditions.

### Comparison with literature

There is a dearth of studies on the effectiveness of telerehabilitation and digital care programs on acute MSK conditions. The pain reduction herein reported (64.3%, with 74.8% participants above MCID of 2.0 [47]) is higher than that reported by Huber et al.*,* [61] for an app-based intervention for low back pain (LBP) including education, physiotherapy and mindfulness (44%, from 4.5 to 2.5 points), as well as that reported by Sandal et al., [62] after an intervention that combined usual care with a self-management LBP app including exercises and educational articles (33%, from 4.9 to 3.3). These differences may reflect the cohorts used by these studies, that included participants with both acute and chronic LBP. Additionally, the DCP presented herein includes biofeedback allied to continuous monitoring by a physical therapist, which might have also contributed for the observed changes.

Pain reductions observed in this study were higher than those reported in papers detailing other non-digital interventions [63–65]. A study on conventional therapy for shoulder pain [65] reported pain reductions of 60.0% (vs 64.3% in this study). In a study on exercise versus cognitive interventions in subacute low back pain, the improvement was of 28 and 37.5%, respectively [63], much lower than that reported herein. Importantly, a control group receiving usual care (i.e management by a primary care physician) showed the worst improvement (17%). Indeed, the combination of both exercise and cognitive interventions in this DCP may explain the greater improvement, in agreement with the findings of Campello et al.*,* [64] who reported a 47.5% improvement in NPRS in subacute low back pain with exercise plus CBT.

As noted above, we observed clinically meaningful improvements in both anxiety and depression, with reductions in GAD-7 (− 5.04) and PHQ-9 (− 5.64) higher than the MCID for both conditions (3.8 and 5, respectively) [66, 67]. Our results reinforce the need to address mental health as part of the intervention, especially since they can be barriers to treatment engagement [68, 69], as also denoted here. Moreover, the correlation between PHQ-9 reduction and pain improvement suggests that MSK pain can be a causal factor for depression [70], that depressive symptoms can result in pain amplification and reduced tolerance (supported by our findings of heightened pain during sessions), or both.

Additionally, influencing fear-avoidant behaviors has been reported as a tool to prevent pain from becoming chronic [8, 12, 71, 72]. The reductions herein observed for FABQ-PA (39.5%) are slightly lower that those reported by Storheim et al.*,* [63] and Campello et al.*,* [64] for a combination of exercise and CBT in subacute low back pain (59.9% improvement), but baseline values were lower in our sample (10.9 vs 14.2).

Regarding productivity, and contrary to what we observed, two previous RCTs failed to observe improvements in this domain after exercise interventions [73, 74], which may be associated with the lack of a biopsychosocial approach such as that featured in this DCP. Very high recovery in productivity (79.0% reduction in overall productivity impairment and 81.4% in presenteeism), was correlated with anxiety reduction and also pain reduction, in line with the findings of Cochrane et al.*,* [75].

### Completion and engagement

Patient engagement has been one of the main challenges in delivering effective home-based MSK care [76]. In this study, we observed a completion rate of 87.5%, higher than that reported for conventional or home-based rehabilitations (range 30–76.3%) [34, 77, 78], and by other digital MSK interventions (73–87%) [62, 79].

We also observed high engagement, with subjects completing average 3.2 sessions per week (SD 1.29). Engagement was higher in the first weeks, which is similar to what has been reported by other authors [76], and may help explain the steeper reduction in pain and secondary outcomes early on. While comparison of these results with published literature is difficult, they are higher than those reported by Guillodo et al. [80] in a study on rehabilitation after ankle sprains, and similar to those reported for digital MSK interventions [79].

### Strengths and limitations

This is the first longitudinal study demonstrating the feasibility of a DCP for patients with acute MSK conditions involving several body areas. The major strengths of this study are the large sample size, the wide range of MSK conditions studied, the breadth of outcomes measured, and the very high retention rate and adherence level. The latter is particularly important considering low adherence is a major barrier to maximizing recovery [81]. In line with current evidence, the digital format, providing flexibility and convenience for participants, may have been key to enhance adherence [76].

The intervention herein presented consisted of different components, namely exercise and education, including CBT-like modules. A biopsychosocial approach such as this program provides is strongly recommended in the management of MSK conditions [19, 82, 83]. Supervision and communication between the patients and PT may also have contributed to our observed results [84]. This approach, however, did not allow us to assess the individual importance of each component.

The limitations of this study are mainly related to study design. This was a prospective single-arm study and did not include a control group. Given the real-world context in which this was performed, the most obvious comparator group would be a “waiting list” control, which we did not have given the high accessibility this technology affords. Finally, a longer follow-up could have provided data on full recovery and re-injury rates.

### Future perspectives

The large sample size and the multiple outcomes assessed provided important insights regarding the feasibility and scalability of the DCP program. These will guide the planning of future RCTs to determine the extent of the impact of the DCP in comparison with conventional therapies or other digital programs. By including specific functional outcomes, and long-term assessment timepoints it will be possible to evaluate full recovery and re-injury rates, as well as to potentially identify treatment response clusters.

## Conclusions

This study provided important insights regarding the feasibility and scalability of the DCP program in a real-world context. Very high completion and engagement rates were observed, as well as significant reductions in pain, medication intake, surgery intent and significant changes in mental health and fear-avoidant behaviors. These changes were associated with marked productivity recovery. We believe this DCP can represent a step further in the delivery of effective and scalable MSK care, and that it holds great promise as a relevant contribution to easing the burden of MSK conditions.

## Supplementary Information

**Additional file 1.** Acute dataset.

**Additional file 2: Supplementary Figure 1.** Example path diagram for the LGC models used in the current study. LGCs are a form of structural equation model for modeling longitudinal processes. Squares represent outcome variables at baseline, 4 weeks, 8 weeks, and 12 weeks. Circles represent average latent intercept (I), slope (S), and curvature (C) components across all participants. Single-headed arrows represent fixed loadings relating the outcome variables to the latent components. Double-headed arrows represent either variance, covariance, or error variance parameters.

**Additional file 3: Supplementary Figure 2.** Distribution of acute MSK conditions per program timepoint.

**Additional file 4: Supplementary Table S1.** Baseline characteristics of Completers and Non-completers.

**Additional file 5: Supplementary Table S2.** Baseline characteristics of 8 weeks and 12 weeks Completers.

**Additional file 6: Supplementary Table S3.** Model fit assessment of the unconditional filtered and unfiltered Latent Growth Curve analysis.

**Additional file 7: Supplementary Table S4.** Filtered and unfiltered conditional Latent Growth Curve Models, assessing body mass index, age and sex as covariates.

**Additional file 8: Supplementary Table S5.** Baseline characteristics of responders and non-responders among completers with available scores (*N* = 250).

**Additional file 9: Supplementary Table S6.** Usability and engagement for completers with available scores with mental health comorbidities.

## Data Availability

All data relevant to the study are included in the article or are available as Digital Content at supplementary material. Only de-identified individual participant data is provided. Further information, including the study protocol, can be found at ClinicalTrials.gov (NCT04092946).

## Abbreviations

ANOVA: Analysis of Variance
BMI: Body Mass Index
CBT: Cognitive Behavioral Therapy
DCP: Digital Care Program
FABQ-PA: Fear-Avoidance Beliefs Questionnaire for physical activity
GAD-7: Generalized Anxiety Disorder 7-item questionnaire
IRB: Independent Review Board
LGCA: Latent Growth Curve Analysis
MCID: Minimal Clinically Important Difference
MSK: musculoskeletal
NPRS: Numerical Pain Rating Scale
PHQ-9: Patient Health 9-item questionnaire
PT: Physical Therapist
RCT: Randomized Controlled Trial
SD: Standard Deviation
WPAI: Work Productivity and Activity Impairment questionnaire
