# Supplementary material for: Telerehabilitation of acute musculoskeletal multi-disorders: prospective, single-arm, interventional study
Source: BMC Musculoskelet Disord. 2022 Jan 4;23:29. doi: 10.1186/s12891-021-04891-5 (PMC8728982; doi:10.1186/s12891-021-04891-5)
Supplement: Supplementary file 2 — Additional file 2: Supplementary Figure 1. Example path diagram for the LGC models used in the current study. LGCs are a form of structural equation model for modeling longitudinal processes. Squares represent outcome variables at baseline, 4 weeks, 8 weeks, and 12 weeks. Circles represent average latent intercept (I), slope (S), and curvature (C) components across all participants. Single-headed arrows represent fixed loadings relating the outcome variables to the latent components. Double-headed arrows represent either variance, covariance, or error variance parameters. [file 12891_2021_4891_MOESM2_ESM.pdf]

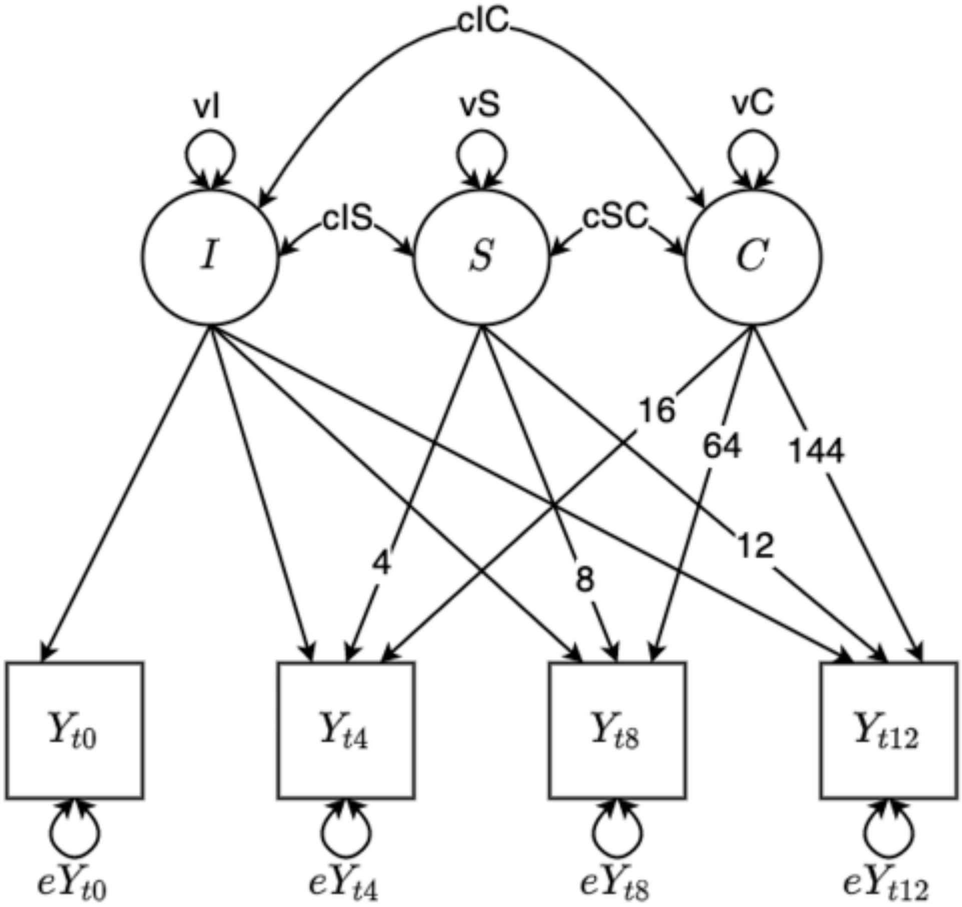

Supplementary Figure 1. Example path diagram for the LGC models used in the current study. LGCs are a form of structural equation model for modeling longitudinal processes. Squares represent outcome variables at baseline, 4 weeks, 8 weeks, and 12 weeks. Circles represent average latent intercept (I), slope (S), and curvature (C) components across all participants. Single-headed arrows represent fixed loadings relating the outcome variables to the latent components. Double-headed arrows represent either variance, covariance, or error variance parameters.

Latent growth curve (LGC) models take the form:

$$Y_{ij} = I + St_i + Ct_i + u_{Ij} + u_{sj}t_i + u_{cj}t_i + e_{ij}$$

where Y is the outcome score for person, j, at time, i, t_i is a vector of time points representing [0, 4, 8, 12] weeks, I is an intercept term representing the average value at $t=0$ for all

participants, S is a slope term representing the average linear change of Y_{ij} over time for all participants, C is a curvature term representing the quadratic change over time of Y_{ij} for all participants, u_{ij} is a random intercept term representing individual participant variation in I, u_{sj} is a random slope term representing individual participant variation in S, u_{cj} is a random curvature term representing individual participant variation in C, and e_{ij} is an error term (see Supplementary Figure 1).
