# Supplementary figures and images for: Telerehabilitation of acute musculoskeletal multi-disorders: prospective, single-arm, interventional study
Source: BMC Musculoskelet Disord. 2022 Jan 4;23:29. doi: 10.1186/s12891-021-04891-5 (PMC8728982; doi:10.1186/s12891-021-04891-5)

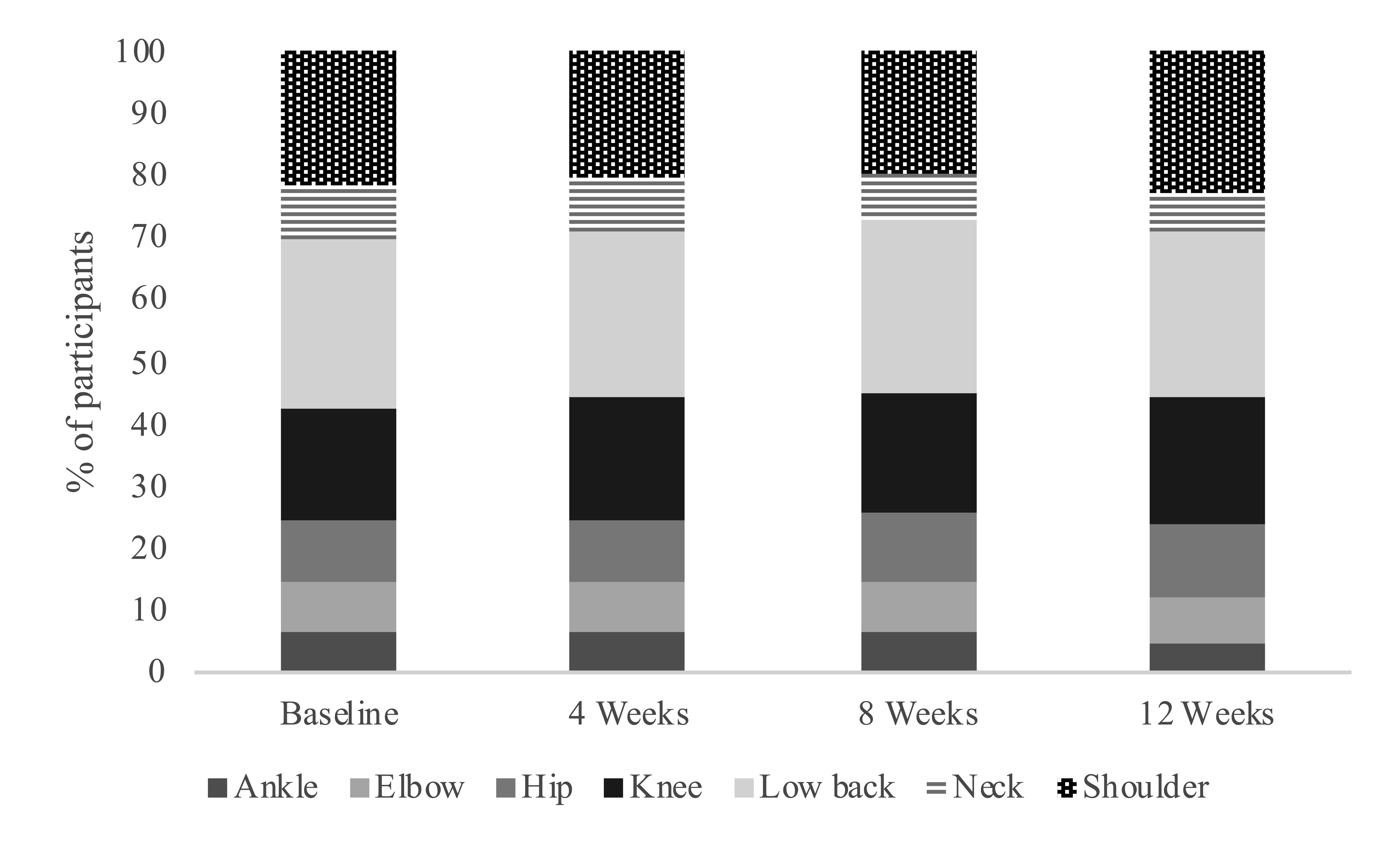

Supplement: Supplementary file 3 — Additional file 3: Supplementary Figure 2. Distribution of acute MSK conditions per program timepoint. [file 12891_2021_4891_MOESM3_ESM.tiff]
