# Supplementary material for: Telerehabilitation of acute musculoskeletal multi-disorders: prospective, single-arm, interventional study
Source: BMC Musculoskelet Disord. 2022 Jan 4;23:29. doi: 10.1186/s12891-021-04891-5 (PMC8728982; doi:10.1186/s12891-021-04891-5)
Supplement: Supplementary file 4 — Additional file 4: Supplementary Table S1. Baseline characteristics of Completers and Non-completers. [file 12891_2021_4891_MOESM4_ESM.docx]

*Supplementary Table S1*

*Baseline characteristics of Completers and Non-completers.*

| **Characteristic** | **Completers (N=300)** | **Non-completers**  **(N=41)** | **p** |
| --- | --- | --- | --- |
| **Age (years), mean (SD)** | 51.3 (11.3) | 49.3 (11.7) | 0.273 |
| **Age categories, N (%):** |  |  | 0.345 |
| - **<25** | 5 (1.7) | 2 (4.7) |  |
| - **25-40** | 58 (19.3) | 7 (16.3) |  |
| - **40-60** | 164 (54.7) | 27 (62.8) |  |
| - **> 60** | 73 (24.3) | 7 (16.3) |  |
| **Sex, Female, N (%)** | 181 (60.3) | 24 (55.8) | 0.572 |
| **BMI, mean (SD)** | 28.7 (6.5) | 29.1 (7.0) | 0.707 |
| **BMI categories, N (%):** |  |  | 0.969 |
| - **Underweight (<18.5)** | 2 (0.7) | 0 (0.0) |  |
| - **Normal (18.5-25)** | 99 (33.0) | 14 (32.6) |  |
| - **Overweight (25-30)** | 99 (33.0) | 15 (34.9) |  |
| - **Obese (30-40)** | 77 (25.7) | 10 (23.3) |  |
| - **Obese grade III (>40)** | 23 (7.7) | 4 (9.3) |  |
| **Education level^#$^, N (%):** |  |  | 0.951 |
| - **High** | 250 (83.3) | 35 (81.4) |  |
| - **Low** | 25 (8.3) | 4 (9.3) |  |
| - **Undisclosed** | 25 (8.3) | 4 (9.3) |  |
| **Employment status^$^, N (%):** |  |  | 0.285 |
| - **Employed**   (part-time or full-time) | 261 (87.0) | 41 (95.3) |  |
| - **Unemployed/Retired** | 39 (13.0) | 2 (4.7) |  |
| **Occupation type, N (%):** |  |  | 0.442 |
| - **White collar** | 258 (86.0) | 40 (93.0) |  |
| - **Blue collar** | 27 (9.0) | 2 (4.7) |  |
| - **Other** (e.g. retired) | 15 (5.0) | 1 (2.3) |  |
| **Body Area, N (%):** |  |  | 0.354 |
| - **Ankle** | 19 (6.3) | 4 (9.3) |  |
| - **Elbow** | 25 (8.3) | 2 (4.7) |  |
| - **Hip** | 33 (11.0) | 2 (4.7) |  |
| - **Knee** | 57 (19.0) | 4 (9.3) |  |
| - **Low back** | 79 (26.3) | 15 (34.9) |  |
| - **Neck** | 26 (8.7) | 4 (9.3) |  |
| - **Shoulder** | 61 (20.3) | 12 (27.9) |  |
| **Exercise level^$, (^days per week), N%:** |  |  | 0.017 |
| - **None** | 41 (13.7) | 13 (30.2) |  |
| - **1-2 days** | 166 (55.3) | 21 (48.8) |  |
| - **3-4 days** | 93 (31.0) | 9 (20.9) |  |
| **Pain Duration^$^, N (%):** |  |  | 0.388 |
| - **< 4 weeks pain** | 92 (30.7) | 16 (37.2) |  |
| - **4-12 weeks pain** | 208 (69.3) | 27 (62.8) |  |
| ^$^ 2 missing values  # High: college/University; Low: professional school or high school; BMI – Body mass index; | | | |
