# Supplementary material for: Telerehabilitation of acute musculoskeletal multi-disorders: prospective, single-arm, interventional study
Source: BMC Musculoskelet Disord. 2022 Jan 4;23:29. doi: 10.1186/s12891-021-04891-5 (PMC8728982; doi:10.1186/s12891-021-04891-5)
Supplement: Supplementary file 5 — Additional file 5: Supplementary Table S2. Baseline characteristics of 8 weeks and 12 weeks Completers. [file 12891_2021_4891_MOESM5_ESM.docx]

*Supplementary Table S2*

*Baseline characteristics of 8 weeks and 12 weeks Completers.*

| **Characteristic** | **8 weeks Completers (N=89)** | **12 weeks Completers (N=161)** | **p** |
| --- | --- | --- | --- |
| **Age (years), mean (SD)** | 50.1 (11.4) | 49.5 (11.3) | 0.262 |
| **Age categories, N (%):** |  |  | 0.791 |
| - **<25** | 2 (2.2) | 1 (1.9) |  |
| - **25-40** | 18 (20.2) | 12 (22.2) |  |
| - **40-60** | 54 (60.7) | 31 (57.4) |  |
| - **> 60** | 15 (16.9) | 10 (18.5) |  |
| **Sex, Female, N (%)** | 53 (59.6) | 29 (53.7) | 0.322 |
| **BMI, mean (SD)** | 28.6 (6.3) | 28.8(6.9) | 0.988 |
| **BMI categories, N(%):** |  |  | 0.982 |
| - **Underweight (<18.5)** | 1 (1.1) | 0 (0.0) |  |
| - **Normal (18.5-25)** | 27 (30.3) | 18 (33.3) |  |
| - **Overweight (25-30)** | 29 (32.6) | 18 (33.3) |  |
| - **Obese (30-40)** | 26 (29.2) | 14 (25.9) |  |
| - **Obese grade III (>40)** | 6 (6.7) | 4 (7.4) |  |
| **Education level^#^, N (%):** |  |  | 0.918 |
| - **High** | 71 (79.8) | 44 (81.5) |  |
| - **Low** | 11 (12.4) | 5 (9.3) |  |
| - **Undisclosed** | 7 (7.9) | 5 (9.3) |  |
| **Employment status, N (%):** |  |  | 0.128 |
| - **Employed**   (part-time or full-time) | 82 (92.1) | 51 (94.4) |  |
| - **Unemployed/Retired** | 7 (7.9) | 3 (5.6) |  |
| **Occupation type, N (%):** |  |  | 0.916 |
| - **White collar** | 81 (91.0) | 137 (85.1) |  |
| - **Blue collar** | 6 (6.7) | 14 (8.7) |  |
| - **Other (e.g. retired)** | 2 (2.2) | 10 (6.2) |  |
| **Affected joint, N (%):** |  |  | 0.206 |
| - **Ankle** | 7 (7.9) | 3 (5.6) |  |
| - **Elbow** | 8 (9.0) | 3 (5.6) |  |
| - **Hip** | 8 (9.0) | 4 (7.4) |  |
| - **Knee** | 18 (20.2) | 4 (7.4) |  |
| - **Low back** | 26 (29.2) | 19 (35.2) |  |
| - **Neck** | 8 (9.0) | 5 (9.3) |  |
| - **Shoulder** | 14 (15.7) | 16 (29.6) |  |
| **Exercise level (days per week),**  **N (%):** |  |  | 0.047 |
| - **None** | 12 (13.5) | 14 (25.9) |  |
| - **1-2 days** | 48 (53.9) | 29 (53.7) |  |
| - **3-4 days** | 29 (32.6) | 11 (20.4) |  |
| **Pain Duration, N (%):** |  |  | 0.524 |
| - **< 4 weeks pain** | 61 (68.5) | 19 (35.2) |  |
| - **4-12 weeks pain** | 28 (31.5) | 35 (64.8) |  |
