# Supplementary material for: Telerehabilitation of acute musculoskeletal multi-disorders: prospective, single-arm, interventional study
Source: BMC Musculoskelet Disord. 2022 Jan 4;23:29. doi: 10.1186/s12891-021-04891-5 (PMC8728982; doi:10.1186/s12891-021-04891-5)
Supplement: Supplementary file 6 — Additional file 6: Supplementary Table S3. Model fit assessment of the unconditional filtered and unfiltered Latent Growth Curve analysis. [file 12891_2021_4891_MOESM6_ESM.docx]

*Supplementary Table S3*

*Model fit assessment of the unconditional filtered and unfiltered Latent Growth Curve analysis.*

| **Outcome** | **Fit** | | | | |
| --- | --- | --- | --- | --- | --- |
|  | **Chi-sq (1)** | ***p*** | **RMSEA** | **CFI** | **SRMR** |
| **Pain** | 30.88 | <.001 | 0.32 | 0.79 | 0.07 |
| **Medication Usage >0** | 0.12 | 0.73 | 0 | 1 | 0.009 |
| **Medication Usage (all)** | 3.31 | 0.069 | 0.09 | 0.96 | 0.03 |
| **Surgery Intent >0** | 3.32 | 0.068 | 0.16 | 0.96 | 0.04 |
| **Surgery Intent (all)** | 4.18 | 0.041 | 0.1 | 0.98 | 0.03 |
| **GAD ≥5** | 2.5 | 0.11 | 0.18 | 0.97 | 0.06 |
| **GAD (all)** | 1.69 | 0.19 | 0.05 | 1,000 | 0.01 |
| **PHQ ≥5** | 1.47 | 0.23 | 0.12 | 0.99 | 0.04 |
| **PHQ (all)** | 1.18 | 0.28 | 0.02 | 1,000 | 0.009 |
| **FABQ** | 5.59 | 0.018 | 0.12 | 0.98 | 0.03 |
| **WPAI Overall >0** | 3.13 | 0.077 | 0.14 | 0.88 | 0.04 |
| **WPAI Overall (all)** | 2.17 | 0.14 | 0.07 | 0.99 | 0.02 |
| **WPAI Work Impairment >0** | 1.95 | 0.16 | 0.09 | 0.95 | 0.03 |
| **WPAI Work Impairment (all)** | 0.75 | 0.39 | 0,000 | 1,000 | 0.01 |
| **WPAI Activity Impairment >0** | 7.97 | 0.0048 | 0.17 | 0.91 | 0.04 |
| **WPAI Activity Impairment (all)** | 6.45 | 0.011 | 0.13 | 0.96 | 0.03 |
| Note: *If a significant chi-square is found for a model, then CFI values > .9, or RMSEA values < .08, or SRMR values <.08 signify models with acceptable fit ^76,77^.* | | | | | |
