# Supplementary material for: Telerehabilitation of acute musculoskeletal multi-disorders: prospective, single-arm, interventional study
Source: BMC Musculoskelet Disord. 2022 Jan 4;23:29. doi: 10.1186/s12891-021-04891-5 (PMC8728982; doi:10.1186/s12891-021-04891-5)
Supplement: Supplementary file 7 — Additional file 7: Supplementary Table S4. Filtered and unfiltered conditional Latent Growth Curve Models, assessing body mass index, age and sex as covariates. [file 12891_2021_4891_MOESM7_ESM.docx]

*Supplementary Table S4*

*Filtered and unfiltered conditional Latent Growth Curve Models, assessing body mass index, age and sex as covariates.*

|  | **BMI** | | | **Age** | | | **Female** | | | |
| --- | --- | --- | --- | --- | --- | --- | --- | --- | --- | --- |
| **Outcome** | **Intercept** | **Slope** | **Curve** | **Intercept** | **Slope** | **Curve** | **Intercept** | **Slope** | **Curve** | |
| **Pain** | 0.01 (0.552) | 0.00 (0.595) | 0.00 (0.871) | 0.01 (0.306) | 0.00 (0.622) | 0.00 (0.913) | **0.42 (0.044)** | -0.12 (0.107) | 0.01 (0.140) | |
| **Medication Usage >0** | -0.02 (0.726) | 0.03 (0.141) | 0.00 (0.126) | -0.01 (0.793) | -0.01 (0.528) | 0.00 (0.312) | 0.43 (0.575) | -0.09 (0.783) | 0.00 (0.951) | |
| **Medication Usage (all)** | 0.03 (0.265) | 0.01 (0.482) | 0.00 (0.333) | 0.02 (0.290) | 0.00 (0.979) | 0.00 (0.902) | 0.42 (0.319) | 0.00 (0.986) | 0.00 (0.863) | |
| **Surgery Intent >0** | **1.03 (0.001)** | **-0.29 (0.030)** | 0.02 (0.066) | 0.18 (0.350) | -0.06 (0.181) | 0.01 (0.121) | -4.59 (0.230) | 0.73 (0.515) | -0.03 (0.733) | |
| **Surgery Intent (all)** | **0.38 (0.008)** | -0.09 (0.105) | 0.01 (0.204) | 0.04 (0.605) | -0.01 (0.519) | 0.00 (0.303) | -2.74 (0.101) | 0.31 (0.491) | 0.00 (0.947) | |
| **GAD ≥5** | -0.07 (0.438) | 0.01 (0.769) | 0.00 (0.825) | 0.04 (0.306) | -0.01 (0.348) | 0.00 (0.973) | -0.01 (0.990) | -0.15 (0.680) | 0.00 (0.979) | |
| **GAD (all)** | 0.02 (0.638) | 0.01 (0.246) | 0.00 (0.176) | **-0.04 (0.013)** | 0.01 (0.094) | 0.00 (0.074) | **0.93 (0.018)** | **-0.20 (0.026)** | 0.01 (0.097) | |
| **PHQ ≥5** | -0.07 (0.400) | 0.03 (0.301) | 0.00 (0.549) | 0.09 (0.071) | -0.01 (0.631) | 0.00 (0.468) | -0.49 (0.744) | 0.54 (0.217) | -0.06 (0.052) | |
| **PHQ (all)** | **0.09 (0.006)** | **-0.02 (0.036)** | **0.00 (0.043)** | -0.01 (0.549) | 0.01 (0.191) | 0.00 (0.107) | 0.13 (0.721) | -0.02 (0.823) | 0.00 (0.879) | |
| **FABQ** | -0.01 (0.899) | 0.01 (0.722) | 0.00 (0.931) | -0.04 (0.199) | 0.00 (0.607) | 0.00 (0.946) | 0.41 (0.541) | 0.06 (0.755) | -0.01 (0.635) | |
| **WPAI Overall >0** | 0.13 (0.555) | 0.06 (0.643) | 0.00 (0.812) | 0.24 (0.159) | **-0.17 (0.007)** | **0.01 (0.010)** | 2.37 (0.499) | -0.66 (0.601) | 0.02 (0.781) | |
| **WPAI Overall (all)** | 0.22 (0.182) | 0.00 (0.993) | 0.00 (0.922) | -0.03 (0.700) | -0.04 (0.122) | 0.00 (0.072) | 2.03 (0.347) | -0.64 (0.322) | 0.05 (0.289) | |
| **WPAI Work**  **Impairment >0** | 0.08 (0.692) | 0.06 (0.606) | 0.00 (0.791) | 0.21 (0.191) | **-0.15 (0.016)** | **0.01 (0.020)** | 1.97 (0.554) | -0.66 (0.560) | 0.04 (0.608) | |
| **WPAI Work**  **Impairment (all)** | 0.18 (0.216) | 0.01 (0.846) | 0.00 (0.933) | -0.04 (0.609) | -0.03 (0.190) | 0.00 (0.131) | 1.55 (0.442) | -0.55 (0.345) | 0.04 (0.250) | |
| **WPAI Activity Impairment >0** | 0.10 (0.657) | 0.06 (0.527) | 0.00 (0.743) | -0.03 (0.736) | 0.01 (0.785) | 0.00 (0.792) | **7.04 (0.003)** | -1.32 (0.106) | 0.08 (0.214) | |
| **WPAI Activity Impairment (all)** | 0.08 (0.712) | 0.05 (0.483) | 0.00 (0.683) | -0.16 (0.099) | 0.03 (0.445) | 0.00 (0.657) | **6.45 (0.005)** | -1.31 (0.062) | 0.08 (0.141) | |
| Note: All covariate p-values shown in parentheses | | | | | | | | | |  |
