# Supplementary material for: Telerehabilitation of acute musculoskeletal multi-disorders: prospective, single-arm, interventional study
Source: BMC Musculoskelet Disord. 2022 Jan 4;23:29. doi: 10.1186/s12891-021-04891-5 (PMC8728982; doi:10.1186/s12891-021-04891-5)
Supplement: Supplementary file 8 — Additional file 8: Supplementary Table S5. Baseline characteristics of responders and non-responders among completers with available scores (N = 250). [file 12891_2021_4891_MOESM8_ESM.docx]

*Supplementary Table S5*

*Baseline characteristics of responders and non-responders among completers with available scores (N=250).*

| **Characteristic** | **Non-responders**  **(Pain reduction below MCID)** | **Responders**  **(Pain reduction above MCID)** | **p** |
| --- | --- | --- | --- |
| **Number of participants, N (%)** | 63/250 (25.2%) | 187/250 (74.8%) |  |
| **Pain level baseline, mean (SD)** | 3.27 (1.6) | 4.9 (1.6) |  |
| **Age (years), mean (SD)** | 55.5 (9.3) | 50.8 (11.3) | **0.002** |
| **Age categories, N (%):** |  |  | 0.063 |
| - **<25** | 0 (0.0) | 2 (1.1) |  |
| - **25-40** | 5 (7.9) | 40 (21.4) |  |
| - **40-60** | 38 (60.3) | 103 (55.1) |  |
| - **> 60** | 20 (31.7) | 42 (22.5) |  |
| **Sex, Female, N (%)** | 38 (60.3) | 119 (63.6) | 0.637 |
| **BMI, mean (SD)** | 29.9 (7.6) | 28.7 (6.3) | 0.217 |
| **BMI categories, N (%):** |  |  | 0.836 |
| - **Underweight (<18.5)** | 0 (0.0) | 2 (1.1) |  |
| - **Normal (18.5-25)** | 20 (31.7) | 61 (32.6) |  |
| - **Overweight (25-30)** | 20 (31.7) | 61 (32.6) |  |
| - **Obese (30-40)** | 16 (25.4) | 49 (26.2) |  |
| - **Obese grade III (>40)** | 7 (11.1) | 14 (7.5) |  |
| **Education level, N (%):** |  |  | 0.180 |
| - **High** | 56 (88.9) | 151 (80.7) |  |
| - **Low** | 2 (3.2) | 20 (10.7) |  |
| - **Prefers not to answer** | 5 (7.9) | 16 (8.6) |  |
| **Employment status, N (%):** |  |  | 0.683 |
| - **Employed**   (part-time or full-time) | 54 (85.7) | 164 (87.7) |  |
| - **Unemployed/Retired** | 9 (14.3) | 23 (12.3) |  |
| **Occupation type, N (%):** |  |  | 0.845 |
| - **White collar** | 53 (84.1) | 165 (88.2) |  |
| - **Blue collar** | 5 (7.9) | 15 (8.0) |  |
| - **Other** (e.g. retired) | 5 (7.9) | 7 (3.7) |  |
| **Affected joint, N (%):** |  |  | **0.011** |
| **Ankle** | 4 (6.3) | 11 (5.9) |  |
| - **Elbow** | 4 (6.3) | 16 (8.6) |  |
| - **Hip** | 5 (7.9) | 23 (12.3) |  |
| - **Knee** | 14 (22.2) | 35 (18.7) |  |
| - **Low back** | 10 (15.9) | 58 (31.0) |  |
| - **Neck** | 11 (17.5) | 8 (4.3) |  |
| - **Shoulder** | 15 (23.8) | 36 (19.3) |  |
| **Exercise level (days per week), N (%):** |  |  | 0.086 |
| - **None** | 13 (20.6) | 22 (11.8) |  |
| - **1-2 days** | 27 (42.9) | 107 (57.2) |  |
| - **3-4 days** | 23 (36.5) | 58 (31.0) |  |
| **Pain Duration, N (%)** |  |  | 0.451 |
| - **< 4 weeks pain** | 16 (25.4) | 61 (32.6) |  |
| - **4-12 weeks pain** | 47 (74.6) | 126 (67.4) |  |
