# Supplementary material for: Telerehabilitation of acute musculoskeletal multi-disorders: prospective, single-arm, interventional study
Source: BMC Musculoskelet Disord. 2022 Jan 4;23:29. doi: 10.1186/s12891-021-04891-5 (PMC8728982; doi:10.1186/s12891-021-04891-5)
Supplement: Supplementary file 9 — Additional file 9: Supplementary Table S6. Usability and engagement for completers with available scores with mental health comorbidities. [file 12891_2021_4891_MOESM9_ESM.docx]

*Supplementary Table S6*

*Usability and engagement for completers with available scores with mental health comorbidities.*

| **Usability outcomes** | **GAD-7<5 (N=230)** | **GAD-7≥5**  **(N=70)** | **p^#^** | **PHQ-9 <5**  **(N= 255)** | **PHQ-9** ≥**5**  **(N=45)** | **p^#^** |
| --- | --- | --- | --- | --- | --- | --- |
| **Number of sessions per week** | 3.3 (1.33) | 3.05 (1.16) | 0.126 | 3.4 (1.33) | 2.6 (0.86) | **<0.001** |
| **Total number of sessions** | 33.0 (16.65) | 29.1 (13.29) | 0.074 | 33.2 (16.35) | 25.9 (12.13 | **0.004** |
| **Total exercise time**  **(min)** | 544.7 (276.68) | 459.3 (216.18) | **0.008** | 548.9 (270.72) | 388.1 (186.93) | **<0.001** |
| **Pain during sessions**  **(NRS, 0-10)** | 1.3 (0.88) | 1.8 (0.95) | **<0.001** | 1.3 (0.87) | 2.0 (0.96) | **<0.001** |
| **Fatigue during sessions**  **(NRS, 0-10)** | 1.2 (1.07) | 1.36 (1.15) | 0.276 | 1.2 (1.02) | 1.7 (1.32) | **0.015** |
| **Satisfaction (0-10)** | 8.7 (1.26) | 8.6 (1.24) | 0.521 | 9.0 (1.17) | 8.7 (1.27) | 0.210 |
| ^#^Independent samples t-test | | | | | | |
